# Middle Meningeal Artery Embolization for Chronic Subdural Hematoma: An Analysis of 35 Consecutive Patients

**DOI:** 10.7759/cureus.49098

**Published:** 2023-11-20

**Authors:** Salvatore Palumbo, Kimon Bekelis, Symeone Missios, Kristine Szczepanski, Carolann Sutherland, Patricia Eckardt

**Affiliations:** 1 Neurosurgery, Good Samaritan University Hospital, West Islip, USA; 2 Neurosurgery, Neuroscience Service Line, Catholic Health Services of Long Island, Melville, USA; 3 Neurosurgery, Endovascular Neurosurgery, Good Samaritan University Hospital, West Islip, USA; 4 Nursing, Good Samaritan University Hospital, West Islip, USA

**Keywords:** surgical drainage, recurrent subdural hematoma, minimally invasive treatment, middle meningeal artery embolization, endovascular treatment (evt), chronic subdural hematoma (csdh)

## Abstract

Introduction: There is sufficient scientific literature that demonstrates favorable outcomes using the minimally invasive technique of middle meningeal artery embolization (MMAE) for the treatment of chronic subdural hematomas (cSDH). The authors evaluate the outcomes of 35 consecutive patients treated with MMAE, both with and without adjuvant surgical drainage (ASD), in an attempt to identify variables that may affect the outcome of patients treated with MMAE for cSDH.

Methods: A multivariate retrospective analysis was performed on patients who received MMAE for cSDH, including age, size of cSDH, ASD, presence of unilateral or bilateral collections, and use of anticoagulants.

Results: Twenty patients underwent MMAE with planned ASD, while 15 patients had MMAE only; these groups had an average cSDH size reduction (measured at its greatest width) of 74% and 69% of cSDH, respectively. Of the 15 patients who were initially treated only with MMAE, three (20%) required a rescue craniotomy. Twenty patients (57%) who had initially presented while receiving oral anticoagulants had an average of 71% size reduction with ASD compared to 74% reduction in those without ASD. Patients not using anticoagulants had an 84% and 78% average reduction in size, respectively. Twelve patients presented with bilateral cSDH; patients who received ASD had an average size reduction of 58%; those without ASD had 63%. Patients with unilateral cSDH had 85% and 83% reduction in size, respectively.

Conclusion: Middle meningeal artery embolization, with or without ASD, can be a useful alternative or adjunct to standard surgical treatment for cSDH. Our study found that only three of 35 patients (9%) treated with this method required any further surgical intervention. No patient who received ASD had a recurrence of their cSDH. Further investigation, including prospective randomized studies, would be useful to better identify which patients can benefit and variables that impact the outcome of patients undergoing MMAE.

## Introduction

The treatment of chronic subdural hematomas (cSDHs) can pose a considerable challenge, as this pathology mainly occurs in the elderly, who may present with multiple comorbidities, including the use of anticoagulants. Planning surgical treatment while navigating these risk factors can be difficult. Many patients may not be offered the necessary treatment due to their poor health status. The incidence of cSDH in patients >65 years of age ranges from eight to 19 per 100,000 people [1-3]. Cases in the general population range from one to five per 100,000 [4]. Surgical drainage, including burr holes or craniotomies, has been utilized as the definitive treatment for cSDH. The propensity of these hematomas to recur adds to the challenge of surgical planning and medical management for these patients [5-8]. The reported rates of recurrence following surgical drainage vary from 3% to as high as 37% [9-14]. Because of the significant medical issues potentially precluding elderly patients from surgery as well as the possibility of these hematomas reaccumulating after surgery, middle meningeal artery embolization (MMAE) was introduced as a minimally invasive treatment to potentially mitigate recurrence. This retrospective study reviews 35 consecutive patients treated at our facility with MMAE for cSDH and its efficacy in stabilizing or reducing the size of the hematoma in patients treated non-surgically as well as preventing recurrent hematomas in those who underwent surgical drainage. We also reviewed several variables that may impact its effectiveness [15,16].

It is believed that cSDH develops approximately three weeks or longer following a mild to moderate head injury. The injury initiates a chronic inflammatory reaction, which may or may not initially demonstrate hemorrhage radiographically. Natural pathophysiologic mechanisms will gradually lead to the formation of a neomembrane, which will form around a developing fluid collection in the subdural compartment [17]. This membrane is composed of both an inner and outer layer, which secrete both anti-thrombotic and fibrinolytic enzymes in an attempt to prevent further clot formation and facilitate clot reabsorption [18,19]. This subdural membrane is believed to be the major contributor to cSDH and its pathogenesis. The membrane will form between the dura-arachnoid and meningeal interfaces. This normally exists as only a potential space and is easily separated. A layer of cells that reside at this interface, referred to as dural border cells, is responsible for both phagocytosis and the formation of fibrous connective tissue. These border cells are believed to be responsible for membrane formation [20,21]. The hematoma will initiate within a layer of injured border cells, forcing this potential space to open. The proliferation of epithelial cells from the inner dural surface forms an inner surrounding membrane [17]. Inflammation from the hematoma generates granulation tissue, forming an outer membrane that contains a permeable capillary network [22]. These capillaries have highly permeable endothelial-gap junctions. It is believed exudate from this network can contribute to continued enlargement of the hematoma as well as recurrence [23,24]. Continued formation of thrombin within the hematoma, followed by persistent anticoagulant and fibrinolytic activity, both maintain the membranes and can also generate continued slow expansion of the hematoma [8]. It is hypothesized that eliminating blood supply to the neomembranes using MMAE mitigates or eliminates membrane leakage, allowing for natural reabsorption of the subdural fluid.

## Materials and methods

Study design and setting

The primary purpose of this observational cross-sectional retrospective review was to identify any patient characteristics or variables that may affect the outcomes of patients undergoing MMAE for cSDH. The desired outcome is for patients to require no further surgical intervention for resilient or recurrent hematoma.

After receiving approval from the Good Samaritan University Hospital Office of the Institutional Review Board (IRB#: 2022.10.11.07.06), West Islip, NY, data collection and retrospective review began on all patients who underwent MMA embolization at Good Samaritan University Hospital between May 1, 2019 and December 31, 2022. According to the code of federal regulations for human subjects research, the requirement for informed consent was waived for this retrospective observational study.

Participants and eligibility

**Figure 1:**
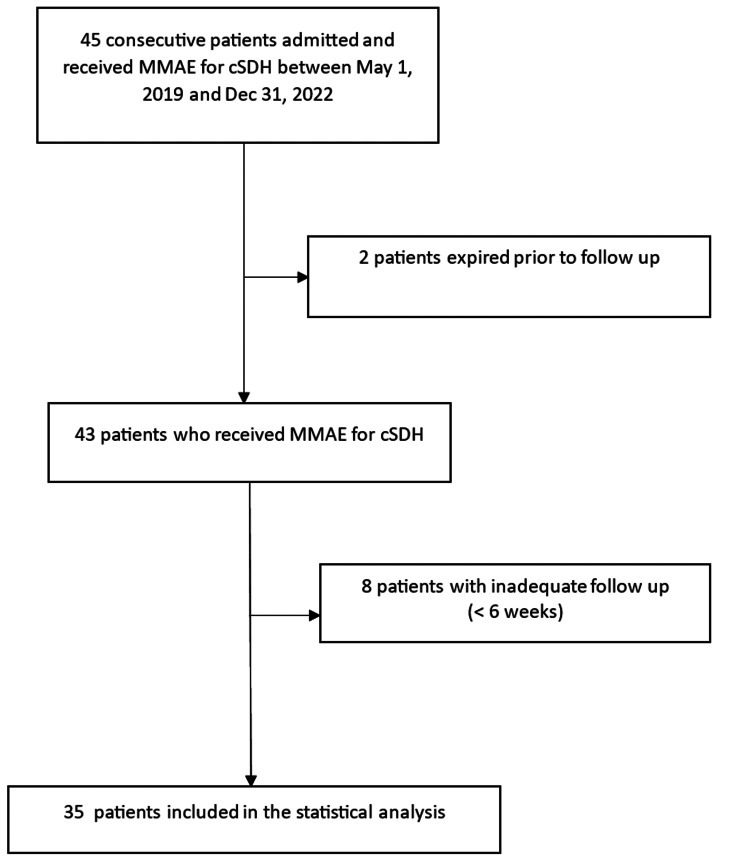
Patient inclusion flowchart MMAE: middle meningeal artery embolization; cSDH: chronic subdural hematoma

The review included patients who presented through the emergency department with cSDH, whether symptomatic or asymptomatic, and had subsequent treatment with MMAE between May 1, 2019, and December 31, 2022. Additionally, patients who were admitted for elective MMAE during the study period were included. Patients were excluded if they had less than six weeks of follow-up or subsequent intracranial procedures not related to the cSDH (Figure 1).

Sample size

A total of 35 patients (Table 1) were included, aged 50-89 years, with a mean age of 73 years.

**Table 1:** Patient characteristics and clinical data ASA: acetylsalicylic acid; F10: factor 10

Patient number	Sex	Age (years)	On anticoagulant on admission	Anticoagulant	Pre-embolization size (mm)	Final size (mm)
1	M	64	No	N/A	11	2
2	F	58	No	N/A	18	0
3	M	71	Yes	Plavix; ASA	3.5	2.3
4	M	83	Yes	Warfarin	14	0
5	M	74	No	N/A	12.9	9
6	M	84	No	N/A	13	2.5
7	F	75	No	N/A	15	0
8	M	54	No	N/A	17.5	0
9	F	77	Yes	ASA	20.4	0
10	M	64	Yes	ASA	33	4
11	M	69	No	N/A	11	0
12	F	50	No	N/A	22	0
13	M	82	Yes	ASA	9	6
14	M	69	Yes	F10; ASA	14	7
15	M	84	No	N/A	19	15
16	F	84	Yes	ASA	11	3
17	F	58	No	N/A	7.5	0
18	M	66	No	N/A	16.6	0
19	M	70	Yes	ASA	38.6	16
20	M	79	Yes	F10; Plavix	7	0
21	M	89	Yes	Plavix	23	5
22	M	79	No	N/A	24	12
23	F	85	Yes	F10; ASA	12	4
24	M	81	Yes	Effient	12	0
25	F	84	No	N/A	14	0
26	M	75	No	N/A	25	7
27	M	51	Yes	ASA	21	6
28	M	76	Yes	ASA	16	0
29	M	59	No	N/A	11	0
30	M	80	Yes	Plavix; ASA	17	5
31	F	84	Yes	ASA	18	5
32	M	62	Yes	ASA	11	7
33	F	73	Yes	Plavix; ASA	12	5
34	M	78	Yes	Plavix; ASA	14	6
35	M	70	Yes	Plavix; ASA	25	2

Twenty patients underwent MMAE with planned adjunct surgical drainage (ASD), while 15 patients had MMAE only.

Variables and data collection

Analysis was then performed on multiple variables, including age, initial and final size of hematoma at last follow-up, whether the hematomas were unilateral (UL) or bilateral (BL), need for rescue craniotomy (unplanned surgical drainage after embolization), and use of anticoagulants. Intracranial imaging, including a CT scan or MRI, was reviewed to measure the initial pre-procedure as well as the evolving post-procedure size of the cSDH. The size was measured at the point of greatest width on axial and coronal images. Each measurement was confirmed by at least two board-certified neurosurgeons. The latest recorded imaging (months following embolization) was assigned as the final date and size of the cSDH.

## Results

The original study population consisted of 45 patients; 36 (80%) had presented through the emergency department and were found to have cSDH. These patients underwent MMAE, with or without ASD, on the same admission. All ASDs were performed within three days of MMAE, either before or after. Eight patients had been admitted electively for treatment of known cSDH with MMAE. Two patients had expired during their hospitalization from complications unrelated to the cSDH; another eight patients were eliminated from the study due to a lack of adequate follow-up. Altogether, 35 patients were included in the review, with an average follow-up of 4.4 months (range: six weeks to 12 months). A univariate analysis of the total sample as well as individual treatment groups was performed (Table 2).

**Table 2:** Univariate analysis Univariate analysis of the total sample and treatment group characteristics Significance tests for continuous and categorical variables were a two-tailed independent samples t-test and a cross-tabulation two-tailed chi-square estimate with one degree of freedom (Fisher’s exact estimate for cells with an expected value of five or less), respectively. MMAE: middle meningeal artery embolization; ASD: adjunct surgical drainage; SD: standard deviation; cSDH: chronic subdural hematoma

Variables	Total sample n=35	MMAE & ASD n=20	MMAE only n=15	p-value
Mean age (SD)	73 (10.6)	69.6 (11.5)	76.8 (8.1)	<0.05
Initial size cSDH (mm) at time of procedure (SD)	16.26 (7.1)	19.10 (7.5)	12.47 (4.5)	<0.01
Presence of bilateral cSDH, n (%)	12 (34%)	6 (30%)	6 (40%)	0.54
On anticoagulants upon admission, n (%)	20 (57%)	9 (45%)	11 (73%)	0.094
Pre-embolization size >15 mm, n (%)	16 (46%)	13 (65%)	3 (20%)	0.008
Age >75 years	18 (51%)	9 (49%)	9 (60%)	0.38

Two of the study groups were found to have significant differences in patient populations upon initiation of the review: those who received MMAE with ASD had a mean age of 69.6 years, while those who received only MMAE had a mean age of 76.8 years. Also, those receiving ASD started with an average cSDH size of 19mm, while those with only MMAE had an average size of 16mm.

Twenty-one patients had a follow-up period of three months or more; 14 patients followed up for less than three months (Table 3).

**Table 3:** Length of follow-up (F/U) Comparison of patients who received middle meningeal artery embolization (MMAE) with and without adjunct surgical drainage (ASD) who had less than three months of radiographic F/U and those with greater than three months; values are a percentage of size reduction at the greatest width of the chronic subdural hematoma (cSDH).

Treatment group	F/U <3 months (n)	F/U >3 months (n)	Combined (n)
With ASD	78% (10)	77% (13)	69% (23)
Without ASD	54% (4)	86% (8)	74% (12)

Comprehensively, those who had MMAE with ASD had an average of 74% reduction of cSDH (n=23, range: 21%-100%), compared to 69% average size reduction in those who did not have ASD (n=12, range: 30%-100%). Those patients with greater than three months of follow-up had an average of 77% and 86% reduction in size with and without ASD, respectively. Patients with less than three months had 77% and 53% reduction in size, respectively. There were no patients who required further surgical intervention or treatment for cSDH who underwent both MMAE and ASD (0%, n=23). Three patients who were initially treated with only MMAE required a rescue craniotomy within one month of treatment (20%). All three of these patients were admitted through the emergency department and had been using oral anticoagulants at the time of presentation. (Plavix and aspirin) These patients had cSDH ranging from 9mm to 17mm; their ages ranged from 72 to 82 years.

The average age of our study population was 72.6 years (range: 50-89 years, median age: 75 years). Seventeen patients were under the age of 75. Twelve of these patients had MMAE with ASD and had an average of an 85% reduction in the size of cSDH. Five patients without ASD had an average reduction of 57%. One of these patients required a rescue procedure (Table 4).

**Table 4:** Patient age details Comparison of patients who received middle meningeal artery embolization (MMAE) with and without adjunct surgical drainage (ASD) in those less than and greater than 75 years of age; values are a percentage of size reduction at the greatest width of the chronic subdural hematoma (cSDH).

Treatment group	Age <75 years (n)	Age >75 years (n)
With ASD	85% (12)	69% (11)
Without ASD	57% (5)	88% (7)
Rescue procedure	1	2

Among a total of 18 patients aged 75 years or more; 11 patients with MMAE and ASD had an average of 69% reduction of their cSDH, while those without ASD had 88%. Two of these patients required rescue procedures.

The initial size of the cSDH at its greatest width was observed, and each patient was placed into one of two groups: less than or greater than 15mm. Patients with an initial size of less than 15mm who had MMAE with ASD (n=9, range: 7.5mm-14mm) and those without ASD (n=10, range: 3.5mm-15mm) had an average reduction of 76% and 72% of the initial size of the cSDH, respectively (Table 5).

**Table 5:** Subdural size Comparison of patients who received middle meningeal artery embolization (MMAE) with and without adjunct surgical drainage (ASD) who presented with a chronic subdural hematoma (cSDH) greater than or less than 15mm; values are a percentage of size reduction at the greatest width of the cSDH.

Treatment group	cSDH <15mm (n)	cSDH >15mm (n)
With ASD	76% (9)	78% (14)
Without ASD	72% (10)	89% (2)
Rescue procedure	2	1

Sixteen patients had cSDH greater than 15mm; those who had MMAE with ASD (n=14, range: 16mm-39mm) demonstrated an average reduction of 78%, while patients with MMAE alone (n=2, range: 17mm-23mm) had 89% average reduction.

The distribution of patients who were using oral anticoagulants at the time of their presentation is shown in Table 6.

**Table 6:** With or without anticoagulants Comparison of patients who received middle meningeal artery embolization (MMAE) with and without adjunct surgical drainage (ASD) who initially presented receiving oral anticoagulants and those who did not; values are a percentage of size reduction at the greatest width of the chronic subdural hematoma (cSDH).

Treatment group	No anticoagulant (n)	Taking anticoagulant (n)
With ASD	84% (11)	71% (12)
Without ASD	78% (4)	74% (8)
Rescue procedure	0	3

Twenty patients (57%) were included in this group: five were taking a combination of Plavix (clopidogrel) and aspirin; two were taking Xarelto (rivaroxaban) and aspirin; two were taking Plavix or Effient (prasugrel) alone; and nine were taking aspirin alone; one each was taking Xarelto or warfarin alone. All patients had their oral anticoagulants discontinued within 72 hours before their embolization or surgery. Anticoagulated patients who underwent MMAE realized an average reduction of cSDH of 71% with ASD compared to 74% without ASD. Those patients not on anticoagulants had 84% and 78%, respectively. The three patients who required a rescue craniotomy had arrived and were taking anticoagulants.

We analyzed whether the presence of UL or BL cSDH had a significant impact on the response to MMAE. Twelve patients had presented with BL collections (34%). Seven patients had ASD with an average size reduction of 58%; those without ASD had an average size reduction of 64%. Having BL cSDHs is the only variable with a difference in size reduction that reached statistical significance (p=<0.01) (Table 7).

**Table 7:** Multivariate analysis of response A multivariate analysis of patient characteristics and treatment on the reduction in lesion size. The significance tests for continuous and dichotomous categorical variables were the Pearson correlation and two-tailed independent samples t-test, respectively. Significance was set at a critical alpha of p 0.05 or less; all tests were two-tailed. cSDH: chronic subdural hematoma; MMAE: middle meningeal artery embolization; ASD: adjuvant surgical drainage

Variables	Statistical test	Statistic estimate	p-value
Age	Pearson correlation	r= - 0.240	0.09
Age >75 years	Independent sample t-test	t= -0.17	0.99
Size of the cSDH at the time of procedure	Pearson correlation	r = 0.053	0.76
Presence of bilateral cSDH	Independent sample t-test	t= 3.05	< 0.01
Pre-embolization size > 15mm	Independent sample t-test	t= 0.68	0.50
On anticoagulants upon admission	Independent sample t-test	t= 1.23	0.29
MMAE only or MMAE & ASD	Independent sample t-test	t= 1.15	0.41

Those with only UL lesions realized average reductions of 85% and 83%, respectively. Table 8 demonstrates a further breakdown and analysis of the 12 patients with BL cSDH.

**Table 8:** Unilateral (UL) or bilateral (BL) chronic subdural hematoma (cSDH) Comparison of patients who presented with BL cSDH and underwent either UL or BL middle meningeal artery embolization (MMAE) and had no adjunct surgical drainage (ASD), UL, or BL ASD; values are a percentage of the size reduction at the greatest width of the cSDH.

Patients with BL cSDH	No ASD (n)	UL ASD (n)	BL ASD (n)
UL MMAE	36% (1)	0	58% (1)
BL MMAE	67% (6)	100% (1)	43% (3)

Six patients underwent bilateral MMAE without any ASD. This group had an average reduction of 67%. Three patients with bilateral MMAE had bilateral ASD, with an average reduction of 43%. One of these patients had a rescue craniotomy. One patient had bilateral MMAE with only unilateral ASD; this patient had 100% resolution of both lesions. Only two patients with bilateral cSDH had just unilateral MMAE. One of these patients had a second side attempted but was aborted due to technical difficulty. One of these patients had no ASD; the second patient had bilateral ASD. They realized average reductions of 36% and 58%, respectively.

## Discussion

Clinicians treating patients with cSDH have often faced a somewhat dichotomous clinical course: surgical drainage poses a minimal technical challenge, while prevention of its recurrence can prove vexing. As our population ages and the percentage of those over 65 years old increases, the incidence of cSDH will increase as well. Moreover, the expanding indications and increasing frequency of interventional cardiac and vascular procedures have necessitated a wider use of oral anticoagulants, which can potentially serve to further increase the incidence in the general population.

The elderly patient population will naturally present with frailty and an increased incidence of significant medical comorbidity along with the cSDH. Some patients may not be eligible to undergo the administration of general anesthesia due to a poor medical condition, which precludes them from surgical drainage. There is a bedside drainage procedure called the subdural evacuating port system (SEPS), whereby a twist drill is used to place a small hole in the skull, followed by perforation of the dura matter and the placement of a catheter and drainage port, which obviates the need for anesthesia. This can potentially decompress a cSDH and relieve a mass effect or a significant intracranial pressure issue related to the cSDH. At our institution, we prefer small craniotomies over burr hole drainage when performing ASD if the patient is deemed a candidate for general anesthesia. This allows the surgeon access to and the ability to excise or fenestrate portions of the neomembrane of the cSDH. None of the patients included in this study had a bedside SEPS procedure. As mentioned earlier in this article, the neomembrane of the cSDH is believed to be a key element in the recurrence of this lesion; elimination of the membrane is now more widely believed to be essential in the treatment of this entity.

Middle meningeal artery embolization was introduced as a minimally invasive technique to help prevent the recurrence of cSDH. Earlier, we mentioned reported recurrence rates of greater than 30%. A more widely accepted rate of recurrence is approximately 10% [3,6,9,11]. None of the patients in our study (0%) who underwent MMAE with ASD experienced a recurrent lesion that required further surgical intervention or hospitalization. This is a significant improvement compared to recurrence rates in historical controls. Using MMAE as a primary treatment without ASD was also highly effective in reducing, and at times, completely eliminating, the volume of cSDH in this study.

The primary outcome of “stand-alone” MMAE (without ASD) is to avoid a surgical drainage procedure in the near or distant future. We performed stand-alone MMAE on patients found to have cSDH and minimal to no symptoms related to the lesion. Three of the 15 patients (20%) who were initially treated with stand-alone MMAE required a rescue craniotomy within one month of the procedure. As mentioned above, all three of these patients had arrived taking oral anticoagulants. Their anticoagulants were discontinued at least 72 hours prior to their MMAE; none of the patients had yet restarted their medication by the time of the craniotomy. Following their rescue craniotomies, they also had somewhat less volume reduction at the last follow-up (58%, 71%, and 33%) compared to the rest of the study population (82%). Their initial subdural collections measured 11mm, 17mm, and 9mm, and their ages were 73, 80, and 82 years, respectively. Comprehensively, patients receiving oral anticoagulants had volume reductions not statistically different than those not taking anticoagulants (p=.094). We cannot say with certainty why these three patients had an inadequate response to MMAE and progressed to surgery. Whether the anti-platelet medication played a role would need to be addressed at a molecular level with neomembrane analysis. We also found that our patients who underwent stand-alone MMAE and had less than three months of follow-up demonstrated a less robust reduction in their volumes (Table 3). This result, however, did not reach statistical significance. This may indicate that slightly more time may be required for the absorption of the hematoma if no surgical drainage is performed. Serial imaging along controlled timelines would be required to address rates of resolution of cSDH following MMAE.

We observed that our patients with bilateral cSDH had a significantly lesser reduction in the size of their lesions when compared to those with unilateral cSDH. The age ranges of these patients were 50-89 years. One can postulate that having bilateral cSDH creates a more significant dead space burden within the subdural space, especially in the elderly population, which already has an element of cerebral atrophy. Having to physiologically re-expand both hemispheres to adequately eliminate the dead space can be problematic, leading to the retention of subdural fluid.

We have reported the average percent reduction of the size of the chronic collection at its greatest width; we found no significant difference in patients having MMAE alone or with ASD. Other studies have reported volume changes associated with MMAE in terms of “less than or greater than”. Schwarz et al. reported their volume changes in terms of greater than or less than 50% [25]. They found that 90% of their patients had volume reductions of greater than 50% in both stand-alone and ASD groups. These findings are similar to ours in that there was no significant difference in volume reduction with or without ASD. They also had three patients who required a rescue craniotomy following a stand-alone MMAE. More importantly, none of the patients had recurrent or residual cSDH following craniotomy, which required any further procedures or hospitalizations.

Our facility has come to find that MMAE has provided significantly improved outcomes to patients presenting with cSDH, used both as a primary, stand-alone treatment or with ASD. Retrospectively, we found 100% of our patients who underwent MMAE with ASD met our primary outcome goal of no significant recurrent or residual cSDH. We also found it effective as a stand-alone treatment, with 80% of these patients able to avoid surgical intervention or further hospitalizations related to the cSDH. We investigated several variables in an attempt to identify which, if any, may impact the effectiveness of MMAE. Although some of these variables demonstrated variance in the percentage of cSDH resolution, ultimately all patients achieved the same desired outcome.

This retrospective study is limited by being a single hospital study with a smaller patient population and small patient numbers within certain sub-groups, limiting statistical power. There are also variations in follow-up, which may alter the final measurements of cSDH. This study is not prospective, lacks randomization of patients, and has the usual limitations of retrospective studies.

## Conclusions

Since its introduction, the use of MMAE has continuously expanded, and there is a growing body of literature demonstrating its safety and utility in preventing the recurrence of cSDH. This retrospective review again finds an improved outcome in patients with cSDH treated with MMAE when compared to historical recurrence rates. Although some variables were associated with differences in volume reduction of the cSDH, there was no significant difference in recurrence rates. We can make no definitive statements or declarations based on our observations. Further investigation, including randomized, prospective studies, is needed to identify variables that may impact the outcome of this procedure. To date, MMAE remains a highly promising adjunct to the treatment of cSDH.
